# Quality of Life for Elderly Residents in Nursing Homes

**DOI:** 10.5539/gjhs.v8n4p127

**Published:** 2015-07-30

**Authors:** Fereshteh Farzianpour, Abbas Rahimi Foroushani, Abbas Badakhshan, Mahin Gholipour, Esmaeil Hosseinzadeh Roknabadi

**Affiliations:** 1 Department of Health Management and Economics, School of Public Health, Tehran University of Medical Sciences, Tehran, Iran; 2 Department of Epidemiology and Biostatistics, Tehran University of Medical Sciences, Tehran, Iran; 3 Golestan Research Center for Gastroenterology and Hepatology at Golestan University of Medical Sciences in Gorgan, Iran

**Keywords:** Quality of Life, elderly, nursing homes, Questionnaires (SF36) and (CPSC), Iran

## Abstract

**Background and Objectives:**

More than 8% of Iran's populations are elderly. The greatest challenge in this generation is improvement of health and QoL. The main goal of this study was QoL for elderly residents in nursing homes over 65 years in Golestan Province - Iran.

**Methods:**

This research was an analytical cross study. The population society includes the elderly over 65 years in Golestan Province - Iran. The sample size was calculated based on the correlation of 193 elderly men and women. Therefore, if the correlation is 2.0 or greater is statistically significant at 80% and 0.95 confidence.

The needed data collected from two questionnaires Consumer product Safety Commission (CPSC) to assess the QOL of nursing homes and the SF-36 for health QOL the elderly indicators through interviews and observation. The reliability of the CPSC questionnaire was estimated using Cronbach's alpha with a coefficient of 0.838. The SF-36 questionnaire was validated with Cronbach's alpha with a coefficient of 0.95. To analyze data, ANOVA one-way test was used that after investigating homogenization of variances with Levin statistic, if homogenization reported P is rejected, the independent T-test was used to interpret it.

**Results:**

Among QOL dimensions only General Health (GH) status showed a significant association with supporting organizations covering status (P = 0.01). The relationship between QOL with marital status in both genders was observed that the General Health (GH) (P = 0.001), Physical Functioning (PF) P = (0.007) Mobility Restricts (MR) P = (0.002), Emotional Problems (EP) (P = 0.001), vitality (V) (P = 0.001), Mental Health (MH) (P = 0.001) were significantly related.

**Conclusions:**

There was a significant relationship between the Physical Functioning (PF) mean and the mean of other QOL indicators in two groups of male and female (P = 0.007), also the safety of nursing homes just related respectively with residence variable (P = 0.01) and their employment (P = 0.031).

## 1. Background

The world's population is aging (Rakhshani et al., 2014). More than 8% of Iran's populations are elderly (Farzianpour et al., 2015). The greatest challenge in this generation is improvement of health and QoL (Farzianpour et al., 2014). QoL subscales were influenced by different factors including age, gender, and financial status and more importantly by health, education, financial and marital status (Farzianpour et al., 2012) Elderly people's health and QoL, when compared with other age groups, are influenced by several factors such as physical, psychological, social and cultural rights (Farzianpour et al., 2012); so that when considering the elderly's health assessment and promotion, the knowledge of an entire health team shall be also considered since it is important for health professionals to work in multidisciplinary and multidimensional teams. Regarding the elderly care, it is necessary to estimate the maintenance of QoL, so that the whole human aging process is considered always seeking for the possibilities of prevention, maintenance and rehabilitation of their health condition (Farzianpour et al., 2004). The improvement in the living conditions has led to the phenomena of aging in different societies (AK, 2010; Kinsella et al., 2005).

Like other countries, Iran has experienced a shift in the population structure towards aging so that according to statistics in 2011, 8.19% of Iran's Populations were older than 60 years (Statistical Yearbook of Islamic Republic of Iran Management and Planning Organization, 2007). As aging is considered an important social issue worldwide, the biggestchallenge is improving the quality of life (QoL) in the elderly (Vameghi et al., 2007; Malekafzali et al., 2010).

QoL is a multidimensional concept that includes the individual's physical, psychological and social performances (Revicki, 1989). The increase of human's life span emphasis the importance of the health-promoting behavior in maintaining and improving QoL (Lee et al., 2006; Lucile et al., 2005; Resnick et al., 2003).

The World Health Organization has placed an emphasis on the importance of health-promoting behavior as a key strategy for maintaining a good QoL (WHO 2002). Pender et al. classified the health-promoting lifestyle (HPL) into six subcategories of nutrition, physical activities, stress management, health responsibility, interpersonal relations, and spiritual growth (Pender et al., 2005).

In Iran, researches have been conducted on different age groups, except the elderly, regarding the association between HPL and QoL (Shankar et al., 1986; Mohamadian et al., 2011).

In developed countries, the association of one or two subscales of HPL with QoL in the elderly have been investigated, which have mainly dealt with the aspect of nutrition and physical activity and the results have indicated that nutrition and physical activities are effective factors in QoL of the elderly (Sohng et al., 2002; Rejeski et al., 2001; McNaughton et al., 2012; Weiwen et al., 2012).

A study in China indicated that interpersonal relations, spiritual growth, and physical activities were better predictors for QoL in older than 50 years of age retired people (Zhang et al., 2013; Zhang et al., 2013). Despite some similarities between the elderly in Iran and the elderly in developed countries, the cultural, religious, and environmental issues should not be neglected (Sonay, 2012). In Iran, the results of a study by Aghanuri et al. have indicated that the quality of nutrition does not have a significant association with the QoL in the elderly (Aghanuri et al., 2013). On the contrary, researches in developed countries show a significant association between nutrition and QoL in the elderly (Kvamme et al., 2011; Keller et al., 2004; Amarantos et al., 2001). Knowledge about the factors that influence QoL is important as population aging becomes a worldwide reality (Lee et al., 2006).

There is less evidence about the amount of influence of HPL on the health-related QoL (HRQoL) in the Iranian elderly and it should be considered that any designed and executed strategy for health promotion needs evidence-based science, because if there is no awareness and recognition of the status of the society's health factors, health programs may just impose high costs. Hence, the results of this study can help health specialists, managers, and policymakers to design and execute health-promoting strategies based on the evidences, and prepare a basis for further researches in the field of aging. The present study assessed QoL using the SF-36 questionnaire to investigate the effects of factors such as age, sex, education, occupation and residence, married status and supporting organizations covering status. The CPSC questionnaire was used to assess the health of the homes nursing environment for elderly residents and its relationship between QoL.

## 2. Methods

This research was approved by the Vice-Chancellor for Research of Tehran University of Medical Sciences and the Research and Ethics Committee as #19430-123225 on 27 March 2012. The study is an analytical cross-sectional study with descriptive and analytical parts. The population was individuals over 65 years of age in the province of Golestanin Iran. In the first phase, the number of elderly in nursing homes in Golestan province was determined. Next, samples were selected from the population of the nursing homes. For sampling, each nursing home was considered to be a cluster for which simple random sampling was carried out and which were surveyed.

The goal was to investigate the association between QoL scores and satisfaction scores in nursing homes. The sample size was calculated based on a correlation coefficient; a correlation of 0.2 or greater is statistically significant at 80% for power of test at a 95% confidence level. The sample size was calculated as:

r = 0.2; w = 0.203; Z 1-a/2 = 1.96; Z 1-b = 0.84

n = (Z 1-a/2 + Z 1-b) ^2/w^2 + 3 = 199

Since each nursing home was considered to be a cluster and there are only five nursing homes in Golestan province, all were studied; thus, the sample size was multiplied by the ratio of cluster sampling (1.5) and the size of sample was obtained as follows:

n = 199×1.5 = 298.5

Because the study population was limited (n =350), the number of samples was adjusted according to the following formula for a final research population of about 193 elderly individuals attending the Jahandidegan Geriatric Charity Institute in the city of Golestan.


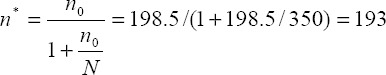


Data was collected using the CPSC questionnaire to measure QoL in nursing homes and the SF36 questionnaire to determine quality indicators. The results were analyzed using SPSS V.17software.

The questionnaires consisted of two parts. The first part obtained demographic and other background information that is assumed to affect QoL and quality indicators of the elderly. The second part contained questions related to QoL. The SF-36 questionnaire scored all questions and the general QoL quantitatively on a scale from 0 to 100. The questionnaires were completed by interview and observation.

The SF-36 consists of 8 dimensions: physical functioning (PF), mobility restriction (MR), bodily pain (BP), general health (GH), vitality (V), social functioning (SF), emotional problems (EP) and mental health (MH). Each dimension was scored from 0 through 100. The SF-36has been validated by Montazeri et al. (2005) as having aCronbach'salpha coefficient of 0.95 for the Iranian population (Montazeri et al., 2005; Mohamadian et al., 2011).

The CPSC questionnaire had 54 items and each item had two options (yes/no). The CPSC has been validated by Farzianpour et al. (2015). The reliability of the CPSC questionnaire was estimated using Cronbach's alpha with a coefficient of 0.838 (Farzianpour et al., 2004, 2015). The chi square test and logistical regression were used to interpret the probability of abnormal QoL between levels of independent predictors. The intra-class correlation coefficient showed that the internal consistency of items on the CPSC was acceptable with an average of 0.815.

Data were analyzed using the chi-square test for one-variable analysis and logistic regression modeling for multi-variable analysis.

**Ethical Considerations**

All participants were given a full explanation of the study and freely consented to participate in the research. The questionnaires did not contain the names of the participants and they were assured that the information collected would be kept confidential and under no circumstances would the published results contain the names of the participants.

## 3. Results

In this study 17.8% of the elderly were men and 82.2 were women. 62% of these people were married, 20.5% of them illiterate. 92.93 percent insurance, 7.1 percent without any support services. In terms of employment of participants also 5.29 percent were unemployed (Table 1).

**Table 1:** Distribution frequency and demographic variables (%) population

variables	N (%)	variables	N (%)
Gender		Education	
male	33(17.8)	illiterate	39(20.5)
Female	152(82.2)	Primary	68(35.8)
Total	185(100)	High school	41(21.6)
Residence		Diploma	34(17.9)
Urban	33(18.6)	Upper Diploma	6(3.2)
Rural	144(81.4)	B.S	2(1.1)
Total	177(100)	Total	190(100)

Marital		Employment	
Single	10(5.4)	Unemployed	4(2.1)
Married	114(62)	Employed	17(1.9)
Divorced	6(3.3)	Fulltime	8(4.2)
Widow	54(29.3)	Part time	9(4.7)
Total support services	184(100)	Housewives	125(66.1)
Relief Committee	11(6)	Farmers	20(10.6)
Welfare	3(1.6)	Retirees	6(3.2)
Health care	68(37)	Total	189(100)
Social security	65(35.3)		
Military	13(7.1)	Total	189(100)

Table 2 showed that among QoL dimensions (PF, MR, EP, V and MH) in both genders, so among QoL dimensions (MR, MH, SF and GH) in both genders of elderly that living in Urban and rural, QoL dimensions (PF, MR, EP, V, BP, GH) for marital status and QoL dimensions (PF, SF, BP, GH) for education status were significantly related (P<0.05). Table 3 showed that association between the CPSC scores and socio-demographical characteristics of the study sample that only variables Residence.

**Table 2:** SF-36 scores versus socio-demographical characteristics of study sample for gender, residence, and marital status

Scales	Sex		P value	Residence		P value	Marital Status mean (SD)				P value
		
	Male mean (SD)	Female mean (SD)		Urban mean (SD)	Rural mean (SD)		Single	Married	Divorced	Widow	
**PF**	62.72(20.84)	50.26(32.34)	0.007	52.67(32.67)	51.6(21.12)	0.866	49(17.28)	6.04(28.54)	58.33(34.59)	36.57(30.12)	0.01
**MR**	63.63(38.57)	38.65(45.74)	0.002	37.32(45.41)	59.0(38.43)	0.006	50(52.70)	53.72(4.40)	45.83(51.03)	20.37(36.68)	0.001
**EP**	82.32(32.79)	57.38(45.32)	0.001	60.04(45.06)	70.7(39.75)	0.213	50(47.49)	47.33(38.77)	80(44.72)	38.36(44.04)	0.001
**V**	66.81(16.28)	56.08(17.66)	0.001	57.12(18.16)	57.62(13.95)	0.883	52.66(9.46)	61.44(18.25)	60(20.49)	51.88(16.75)	0.009
**MH**	68.51(16.15)	57.13(18.06)	0.001	56.38(18.74)	65.75(13.95)	0.01	63.20(20.81)	61.25(17.82)	52.66(34.63)	55.07(16.21)	0.144
**SF**	58.71(15.77)	63.89(26.70)	0.284	65.19(26.28)	54.54(19.96)	0.012	67.50(28.98)	66.66(23.14)	58.33(24.57)	56.48(22.55)	0. 075
**BP**	69.24(13.10)	62.53(22.18)	0.096	63.69(22.13)	65.07(15.92)	0.736	58.50(22.11)	68.53(19.13)	53.75(17.37)	57.26(22.14)	0.004
**GH**	56.51(8.61)	55.51(17.42)	0.749	57.43(16.77)	50.87(12.28)	0.013	48(19.17)	59.68(13.83)	38.33(25.81)	51.29(16.34)	0.001

**Table 3:** SF-36 scores versus socio-demographical characteristics of study sample for Education and Employment

Scales	Education mean (SD)						P value	Employment mean (SD)			
	
	Illiterate	Primary	High school	Diploma	Upper diploma	BS		Unemployed	Employee	Fulltime	Part-time
**PF**	37.82(28.39)	56.25(28.07)	58.53(32.56)	52.18(33.69)	64.16(31.68)	75(14.14)	0.019	51.25(39.66)	51.66(31.75)	68.33(17.13)	55.35(30.02)
**MR**	28.84(39.95)	48.89(44.97)	44.51(48.5)7	41.91(46.35)	58.33(46.54)	25(35.35)	0.303	60(45.41)	33.33(28.86)	52.77(40.39)	50(45.99)
**EP**	50.42(74.06)	70.83(39.93)	55.83(46.15)	63.02(44.34)	83.33(40.82)	1.00(0.00)	0.104	66.66(47.14	66.66(57.73)	90.74(18.83)	80.95(25.19)
**V**	53.84(17.48)	61.76(17.75)	58.3(16.54)	53.57(19.27)	65(17.82)	67.50(24.74)	0.124	66(28.15)	44.44	68.51	55.59
**MH**	60.35(16.26)	61.47(17.43)	56.21(21.93)	54.73(15.79)	71.50(18.19)	60(5.65)	0.207	68.80(18.41)	51.66(7.63)	73.77(10.02)	62.85(17.64)
**SF**	49.35(21.64)	62.68(23.10)	65.54(26.92)	70.22(24.42)	95.83(6.45)	75(35.35)	0.01	70(22.70)	62.50(21.65)	54.16(28.64)	58.03(20.57)
**BP**	55.44(17.71)	67.09(21.89)	62.68(20.94)	65.95(19.69)	78.75(23.86)	77.50(31.81)	0.031	77(15.94)	5.16(18.76)	88.11(12.87)	65.35(12.43)
**GH**	47.53(16.21)	58.75(13.47)	52.43(19.64)	61.02(13.18)	65(7.74)	72.50(17.67)	0.01	62	60	57.63	57.50

Employment were significantly related respectively (P=0.01and p=0.031) Table 4 showed that among QoL dimensions only General Health (GH) status showed a significant association with supporting organizations covering status (P = 0.01). Analysis of the data showed that among QoL dimensions with Physical Functioning (PF) and Mental Health (MH) only (PF) status a significant association with marital status, education respectively (P=0.01and p=0.019) so Mental Health (MH)) status only with current employment status (P=0.005) (Table 4).

**Table 4:** Determinants of physical functioning and mental health related quality of life in elderly participants

Scales	Marital Status	95%CI	P-value
		Lower	Upper	
			
Physical functioning	Single	36.63	61.36	0.01
	Married	54.69	65.39	
	Divorced	22.03	94.63	
	Widow	28.35	44.79	
	Total	47.96	56.86	
Mental Health	Single	48.31	78.08	0.144
	Married	57.94	64.56	
	Divorced	16.32	89.01	
	Widow	50.64	59.50	
	Total	56.59	61.93	
Physical functioning	**Education**			
	Illiterate	28.61	47.02	0.019
					Primary	49.45	63.04
	High school	48.25	68.81	
	Diploma	40.03	64.33	
	Upper diploma	30.91	97.42	
	BS	-52.06	202.06	
	Total	48.23	57.13	

Mental Health	**Education**	2.60	65.62	0.207
	Illiterate	2.11	65.69	
	Primary	3.42	63.14	
	High school	2.70	60.24	
	Diploma	7.42	90.59	
	Upper diploma	4	110.82	
	BS	1.31	61.79	
	Total	1.31	61.79	

Physical functioning	**Employment**			
	Unemployed	-11.85	114.35	
	Employee	-27.21	130.54	
	Fulltime	55.15	81.50	
	Part-time	38.01	72.69	
	Unemployedwith income	-21.52	138.18	
	Housewives	42.55	54.38	
	Farmers	50.41	75.95	
	Retirees	47.63	72.36	
	Total	47.81	56.8

Mentall Health				0.005
	**Employment**			
	Unemployed	45.93	91.66	
	Employee	32.69	70.63	
	Fulltime	66.07	81.48	
	Part-time	52.67	73.04	
	Unemployedwith income	-40.07	125.40	
	Housewives	53.59	59.79	
	Farmers	63.17	83.00		
	Retirees	49.82	68.00		
	Total	56.63	61.86	

General Health	Organizations support services			0.01
	Relief Committee	29.09	72.52	
	Welfare	25.81	77.52	
	Health care	48.91	57.26	
	Social security	56.40	62.48	
	Military	59.18	76.20	
	Insurance Supplementary	48.48	91.51	
	Other	52.74	68.50	
	Total	53.64	58.29	

## 4. Discussion

In general, the obtained scores of SF-36 in this study showed QOL in the elderly were significantly correlated with gender and indicate that males scores higher for QoL than did females. Results of this study were correlated with the more negative attitude of females toward their physical health. The results of the CPSC questionnaire, which was used for the first time in research in Iran, showed that the perception of safety of residence in 98% of cases was moderate (47.7%) or good (50.3%). The study found that the CPSC results correlated significantly with residence for a moderate level of safety for nursing homes in rural areas and a good level of safety in urban areas. CPSC results also showed a significant relationship with level of employment. The results from the CPSC and SF-36 showed a significant relationship only between the CPSC and physical constraints. Our findings support previously described beneficial effects of the counseling model on the elderly QoL in the cities of Tehran (Farzianpour et al., 2004., Rakhshani et al., 2014) Masjed solaiman (Farzianpour et al., 2012), Marivan (Farzianpour et al., 2012), Iran. The mean QoL scores measured in the elderly population, in other countries were much higher than the results obtained in Iran (Tsai et al., 2004). The QoL subscales were influenced by different factors including age, gender, and financial status and more importantly by education, financial and marital status as other studies showed (Farzianpour et al., 2012). Therefore, it is important to inform the elderly population of the behavioral modifications benefits of the QoL. The study of Farzianpour et al also found clear connections between disability and gender and the higher rate of failure in women (Farzianpour et al., 2015).

Gureje and colleagues also point out that the bodily pain which can lead to reduced QoL was higher in women that this issue could be due to a greater incidence of chronic disabling diseases in women than in men (Gureje et al., 2006).

However the study of Farzianpour et al., found that women in vitality and mobility restrict aspects were better than men (Farzianpour et al., 2015).

## 5. Conclusions

It seems that dimensions of QoL are more related to gender and relative superiority of men over women. So the policy makers of health sector should pay more attention to the social factors affecting health such as education, housing and the physical functioning. Particularly that the illiteracy rate among women was higher than men, and also a lot of them were housewives that were an unpaid work and therefore didn’t create any sense of security.

It also seems that due to physiological and anatomical features of women and men, physically activity facilities for women should have provided more and economic and social barriers to be eliminated as soon as possible. It seems that some restrictive measures such as the establishment of special parks in Golestan Province or some other metropolises are not an appropriate strategy to address this deficiency. Relating with different dimensions of (QoL) and age of participants, it is seen that with increasing age of the participants in terms of bodily pain, mobility restricts and mental health increases, this is necessary for authorities to pay attention to these issues and programs to improve their physical activities and specially their mental health.

Overall, this study and other studies suggest that aging alone was not particularly effective on the QoL dimensions especially on mental health and other aspects of life in the elderly.

**Limitations**

The limitations of this study included several changes in management of the State Welfare Organization of the province that delayed the implementation phase of the project. Other restrictions were the lack of cooperation by some elderly for completing the questionnaire and it was necessary to fully explain all options to them. In some cases, educated and interested members of their households were asked to encourage and explain the importance of the project, especially regarding questions on the CPSC questionnaire.
